# HCC Development Is Associated to Peripheral Insulin Resistance in a Mouse Model of NASH

**DOI:** 10.1371/journal.pone.0097136

**Published:** 2014-05-22

**Authors:** Samuele De Minicis, Laura Agostinelli, Chiara Rychlicki, Gian Pio Sorice, Stefania Saccomanno, Cinzia Candelaresi, Andrea Giaccari, Luciano Trozzi, Irene Pierantonelli, Eleonora Mingarelli, Marco Marzioni, Giovanna Muscogiuri, Melania Gaggini, Antonio Benedetti, Amalia Gastaldelli, Maria Guido, Gianluca Svegliati-Baroni

**Affiliations:** 1 Università Politecnica delle Marche, Department of Gastroenterology, Ancona, Italy; 2 Università Cattolica del Sacro Cuore, Policlinico Gemelli, Division of Endocrinology and Metabolic Disease, Rome, Italy; 3 Diabetic Care Clinics, ACISMOM, Rome, Italy; 4 Don Gnocchi Foundation Onlus, Milan, Italy; 5 Institute of Clinical Physiology, Head of Cardiometabolic Risk Unit, Pisa, Italy; 6 Università di Padova, Department of Medicine Anatomic Pathology Unit, Padova, Italy; 7 Università Politecnica delle Marche, Obesity Center, Ancona, Italy; Inserm, U1052, UMR 5286, France

## Abstract

NAFLD is the most common liver disease worldwide but it is the potential evolution to NASH and eventually to hepatocellular carcinoma (HCC), even in the absence of cirrhosis, that makes NAFLD of such clinical importance. Aim: we aimed to create a mouse model reproducing the pathological spectrum of NAFLD and to investigate the role of possible co-factors in promoting HCC. Methods: mice were treated with a choline-deficient L-amino-acid-defined-diet (CDAA) or its control (CSAA diet) and subjected to a low-dose i.p. injection of CCl_4_ or vehicle. Insulin resistance was measured by the euglycemic-hyperinsulinemic clamp method. Steatosis, fibrosis and HCC were evaluated by histological and molecular analysis. Results: CDAA-treated mice showed peripheral insulin resistance at 1 month. At 1–3 months, extensive steatosis and fibrosis were observed in CDAA and CDAA+CCl_4_ groups. At 6 months, equal increase in steatosis and fibrosis was observed between the two groups, together with the appearance of tumor. At 9 months of treatment, the 100% of CDAA+CCl_4_ treated mice revealed tumor versus 40% of CDAA mice. Insulin-like Growth Factor-2 (IGF-2) and Osteopontin (SPP-1) were increased in CDAA mice versus CSAA. Furthermore, Immunostaining for p-AKT, p-c-Myc and Glypican-3 revealed increased positivity in the tumors. Conclusions: the CDAA model promotes the development of HCC from NAFLD-NASH in the presence of insulin resistance but in the absence of cirrhosis. Since this condition is increasingly recognized in humans, our study provides a model that may help understanding mechanisms of carcinogenesis in NAFLD.

## Background

Hepatocellular carcinoma (HCC) is the third most common cause of cancer-related death worldwide. [1], [2]. Moreover, several studies have shown that 5% to 30% of patients with HCC lack a readily identifiable risk factor for their cancer [1]. Most of these ‘‘cryptogenic’’ HCC might be attributed to Non-Alcoholic Fatty Liver Disease (NAFLD) and the concomitant metabolic syndrome [2], [3], nevertheless it is not yet clear what predisposes to the progression of the disease [4].

On this regard, NAFLD is a major health problem that integrates several liver conditions ranging from simple fatty liver to Non-Alcoholic Steatohepatitis (NASH), which is associated with fibrosis that may evolve into cirrhosis and results into HCC [5]. How HCC develops from NASH livers is still obscure. Some hypotheses suggest that obesity, insulin resistance, release of inflammatory cytokines and autophagy can contribute to the carcinogenic potential in NASH liver, where HCC can occur in 65% of patients without an over cirrhosis in the background liver [6]. However, no direct link has been provided yet. Studies that aim to link HCC and NAFLD are blunted by the lack of reliable animal models. The use of a choline-deficient L-aminoacid-defined diet (CDAA) in rats provided the most interesting results inducing steatohepatitis [7]. Concerning this issue, a still unresolved question is however related to the potential of CDAA diet in inducing insulin resistance [8], [9]. The purpose of this study is to develop a mouse model of liver injury which mimics NASH features that lead to HCC.

## Materials and Methods

### Animals and Treatment

Male 6 to 8 weeks old C57BL/6 mice were purchased from Charles River Laboratories International, Inc. (Wilmington, MA). Animals were fed a CDAA diet or its control diet, CSAA diet (Laboratorio Dottori Piccioni, Milano, Italy). In parallel experiments, starting simultaneously to the diet administration, liver fibrosis was enhanced by weekly intraperitoneal injections of Carbon Tetrachloride (CCl_4_) at the dose of 0.2 µl/g of body weight. Mice (8 mice/group) were sacrificed at 1, 3, 6 and 9 months of treatment and samples processed for histopathology, serological and molecular analysis.

All animal work has been conducted according to the local committee for care and use of laboratory animals. The study protocol was approved by the Ethical Committee for animal experimentations of the “Università Politecnica delle Marche” under the protocol named FIRB07/2011GE.

### Evaluation of Peripheral Insulin Resistance

After 1 month of treatment, we evaluated peripheral insulin resistance by the euglycemic-hyperinsulinemic clamp method [10]. Surgery for the positioning of catheters was performed 3 to 5 days prior to the insulin clamp procedure as previously described [11]. The euglycemic-hyperinsulinemic clamp was performed in the awake unrestrained state after 6 hour-fast. At time 0, a primed continuous (4.0 mU/kg/min, Actrapid 100U, Novo Nordisk, Copenhagen, Denmark) infusion of human insulin was started simultaneously with a variable infusion of 10% dextrose in order to maintain the plasma glucose concentration constant at its basal level (80–100 mg/dl). Fasting Plasma Glucose (FPG) was measured at time 0. Subsequently, blood samples were taken from the tail vein at 10 min intervals for at least 2 hours to measure glucose concentration and adjust dextrose infusion rates. Insulin sensitivity was calculated as M value (rate of peripheral glucose uptake) from average glucose concentrations and dextrose infusion rates during the last 30 min of the steady-state clamp period. Fasting insulin concentrations were measured using an ELISA kit (Merck Millipore, Darmstadt, Germany).

### Triglyceride Measurement

Hepatic triglyceride content was measured as previously reported [12].

### Liver Histology and Immunohistochemistry

Mice liver tissues were fixed in 10% buffered formalin and embedded in paraffin. Liver sections were routinely stained Hematoxylin and Eosin and Sirius Red. Frozen sections from the different groups of mice were stained for Oil Red O. Diagnosis of NASH was based on the presence of steatosis, inflammation and hepatocyte ballooning. The severity was assessed by using the NAS scoring system [13].

For Immunohistochemistry, liver sections were incubated with primary monoclonal antibodies against Alpha-Smooth Muscle Actin (αSMA) (DakoCytomation, Carpinteria, CA), p-AKT Ser473, β-Catenin (Cell Signaling, Boston, MA), p-c-Myc Ser373 (Biorbyt, Cambridge, UK) and Glypican-3 (Cell Marque, Rocklin, CA). Antibodies against Ki67 and F4/80 were from ABCAM, Cambridge, UK. In Situ Cell Death (TUNEL) kit was from Roche, Manneheim, Germany. Detection of positive staining was performed by using DAB reagent (Sigma-Aldrich, St. Louis, MO).

Quantitative morphometry was performed as previously described [14], [15].

### Quantitative Real-time Reverse Transcription-Polymerase Chain Reaction (qRT-PCR)

Total RNA was extracted from mice liver using TRIzol Reagent (Life Technologies Corporation, Woburn, MA). Total RNA was reverse-transcribed to complementary DNA [14]. qRT-PCR was performed using Rotor-Gene 6000 instrument (Corbett Life Science Pty. Ltd., Mortlake, NSW, Australia). The relative abundance of the target genes was normalized to 18S rRNA as internal control.

### Statistical Methods

Results are expressed as mean ± SEM. The results were analyzed using ANOVA test. A *p* value of less than 0.05 was considered statistically significant.

## Results

### Treatment with CDAA Induces Insulin Resistance

**Figure 1:**
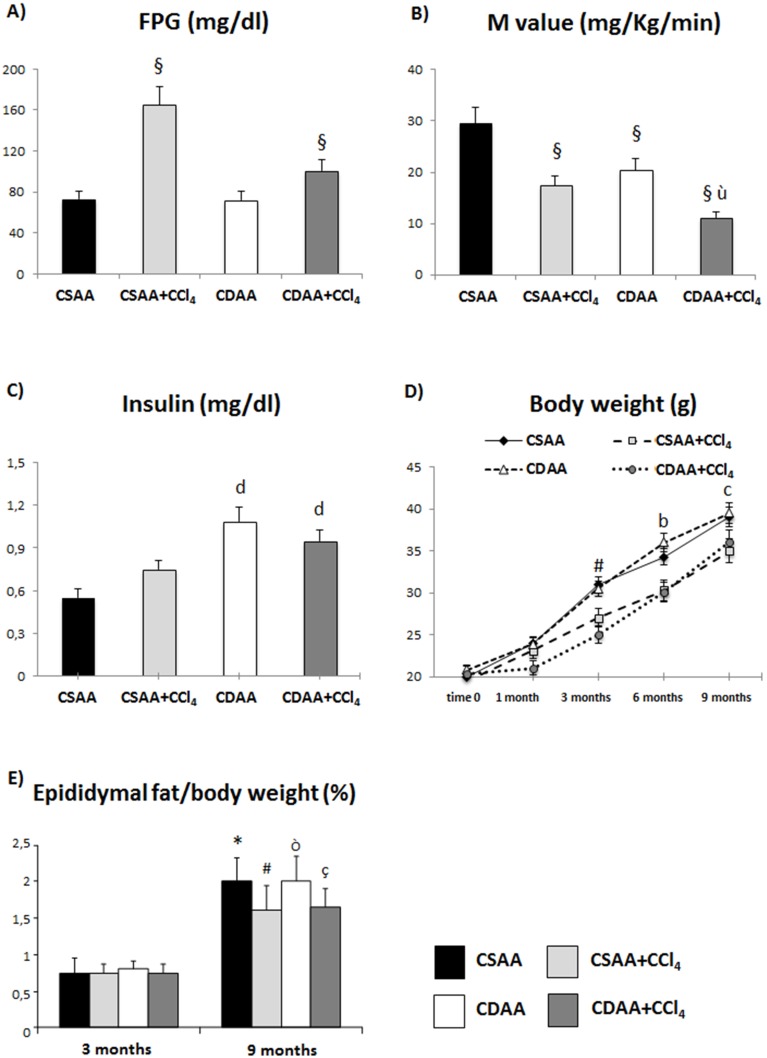
Effect of diet treatment on metabolic parameters and body weight. A–B) Mice treated with CDAA diet for 1 month develop peripheral insulin resistance as shown by the lower glucose uptake vs CSAA-treated mice (M value) with similar levels of FPG. CCl_4_ treatment induces an increase in fasting plasma glucose and enhances the condition of insulin resistance, reducing M value. C) Insulin levels measured at 1 month of treatment. D) The graphic shows a gradual increase in body weight in both CDAA and CSAA diet. CCl_4_ injection similarly reduces body weight in both diets. E) Epididymal fat/body weight ratio appears to be similar between the two different diets both at 3 and 9 months of treatment, with similar reduction in the presence of CCl_4_. Data represent mean ± SD; §p<0.05 vs CSAA; ù p<0.05 vs CDAA; *p<0.05 vs CSAA 3 months; #p<0.05 vs CSAA+CCl_4_ 3 months; ò p<0.05 vs CDAA 3 months; ç p<0.05 vs CDAA+CCl_4_ 3 months; b p<0.05 vs CSAA+CCl_4_ 6 months; c p<0.05 vs CSAA+CCl_4_ 9 months; d p<0.05 vs CSAA.

We evaluated the effects of CSAA and CDAA diets with the association of CCl_4_ on peripheral insulin resistance by the euglycemic-hyperinsulinemic clamp method. After 1 month of treatment, mice treated with CDAA diet develop peripheral insulin resistance as shown higher fasting plasma insulin concentrations (Fig. 1C) and the lower glucose uptake in comparison to CSAA-treated mice (M value, Fig. 1B), despite a similar level of FPG between the two diets evaluated at time 0 (Fig. 1A). Conversely, CCl_4_ treatment induces a significant increase in FPG levels and potentiates the lowering of peripheral glucose uptake both in CSAA and CDAA diets (Fig. 1A–B). The above mentioned experiments were performed by infusing insulin at a very high rate, which is associated to a complete suppression of hepatic glucose production. Insulin levels increased in animals treated with CDAA (Fig. 1C).

Moreover, both CSAA and CDAA diets lead to a progressive and time-dependent increase in body weight (Fig. 1D). This was associated to a constant amount of caloric intake that did not differ between CSAA- and CDAA-treated animals at all time points, not significantly affected by CCl_4_ administration (data not shown). Furthermore, mice treated with a concomitant chronic low-dose injection of CCl_4_ show a significant reduction in body weight in comparison to not injected-mice (Fig. 1D).

The epididymal fat/body weight ratio strongly confirms the previous results (Fig. 1E), showing a significant increase from 3 to 9 months with both diets, with similar reduction in the presence of CCl_4_.

### Treatment with CDAA Determines a Progressive Increase of Hepatic Steatosis

**Figure 2:**
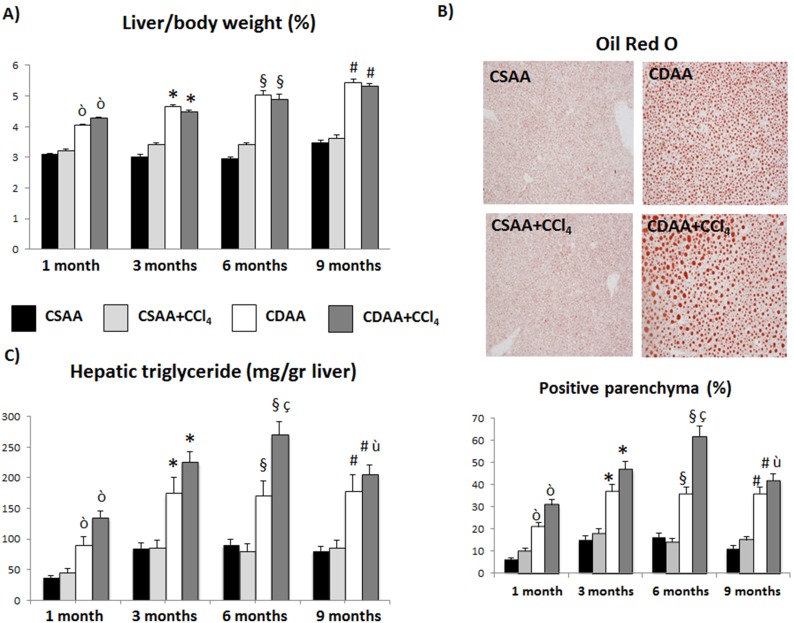
Treatment with CDAA determines a progressive increase of hepatic steatosis. A) Increased levels of liver/body weight fraction are observed in the group of mice treated with CDAA diet, either with or without CCl_4_ administration, in comparison to mice treated with control diet, starting at 1 month. B) Histochemical staining and relative morphometric evaluation for Oil Red O and C) hepatic triglyceride measurement show increased deposition of lipids in the liver parenchyma of CDAA and CDAA+CCl_4_ treated animals in comparison to CSAA treated mice. Data represent mean ± SD; ò p<0.05 vs CSAA 1 month; *p<0.05 vs CSAA 3 months; §p<0.05 vs CSAA 6 months; #p<0.05 vs CSAA 9 months; ç p<0.05 vs CDAA 6 months; ù p<0.05 vs CDAA 9 months.

CDAA diet-treated mice show an increase in liver/body weight ratio (Fig. 2A), compared to CSAA diet, starting already at 1 month. No significant differences are observed in association to CCl_4_.

Oil Red O staining in mice treated with CDAA shows significantly higher lipid deposition in comparison to mice treated with CSAA as can be observed in the representative pictures at 3 months treatment and quantified by the morphometric analysis for each time point (Fig. 2B). Furthermore, hepatic triglyceride content is increased in CDAA- and CDAA+CCl_4_-treated animals but not in its control diet alone or together with CCl_4_ (Fig. 2C).

### Gene Expression Modifications of Enzymes of Carbohydrate and Lipid Metabolism

**Figure 3:**
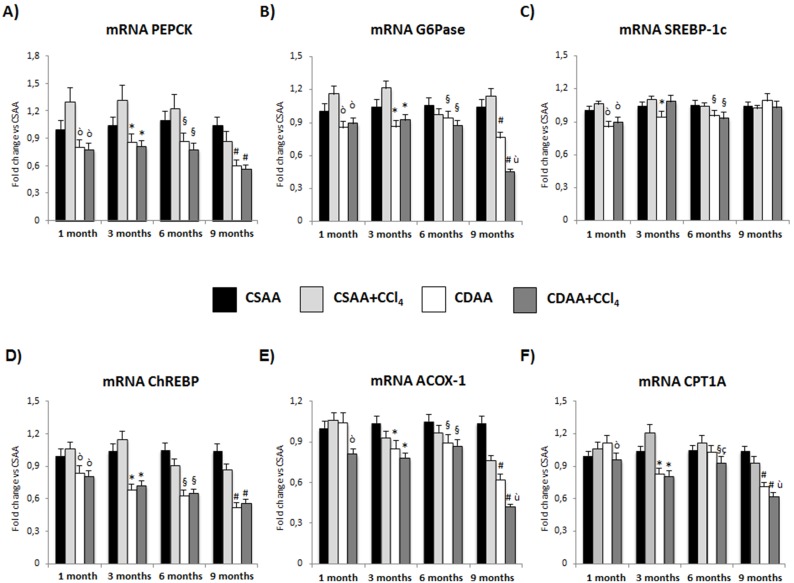
Gene expression of elements involved in carbohydrate and lipid metabolism. qRT-PCR expression shows a progressive decrease of the amount of PEPCK (A), G6Pase (B), ChREBP (D), ACOX-1 (E) and CPT1A (F) mRNA expression in the groups of CDAA or CDAA+ CCl_4_, while only a slight decrease is observed for mRNA expression of SREBP-1c (C). Data represent mean ± SD; ò p<0.05 vs CSAA 1 month; *p<0.05 vs CSAA 3 months; §p<0.05 vs CSAA 6 months; #p<0.05 vs CSAA 9 months; ç p<0.05 vs CDAA 6 months; ù p<0.05 vs CDAA 9 months.

Hepatic steatosis was associated to specific modifications in gene expression of elements involved in carbohydrate and lipid metabolism (Figure 3). The expression of enzymes associated with gluconeogenesis, as Phosphoenolpyruvate Carboxykinase (PEPCK) and glucose production, as Glucose 6-phosphatase (G6PC) was reduced in the liver of CDAA-treated animals starting at 1 months, and further decreased at 9 months, independently from CCl_4_ administration indicating that hepatic insulin resistance was not increased by CDAA (Fig. 3A–B).

On the other hand, the hepatic expression of the Sterol Regulatory Element Binding Protein-1c (SREBP-1c), that is the transcription factor activating all genes required for lipogenesis [16], was slightly reduced by CDAA treatment without major modifications induced by CCl_4_ administration in our experimental model (Fig. 3C). Furthermore, the Carbohydrate-Responsive Element-Binding Protein (ChREBP), a key element of glucose-mediated stimulation of lipogenesis [16] progressively decreased in CDAA-treated animals, independently from CCl_4_ administration, starting at 1 month (Fig. 3D). These data indicate that the development of steatosis in CDAA model was not associated with an increased lipogenesis.

Thus, we analyzed the pathways related to FA oxidation, finding a decreased expression of both ACOX-1 (a rate-limiting enzyme in peroxisomal fatty acids β-oxidation) and CPT1A (a key enzyme in mitochondrial fatty acids β-oxidation) [12] observed in CDAA+CCl_4_-treated animals at 1 months and in CDAA mice thereafter, indicates that reduced fatty acid oxidation could be one of the mechanisms of hepatic steatosis (Fig. 3E–F).

### Treatment with CDAA or CDAA+CCL_4_ Induces Liver Damage and Hepatic Fibrosis in Mice

**Figure 4:**
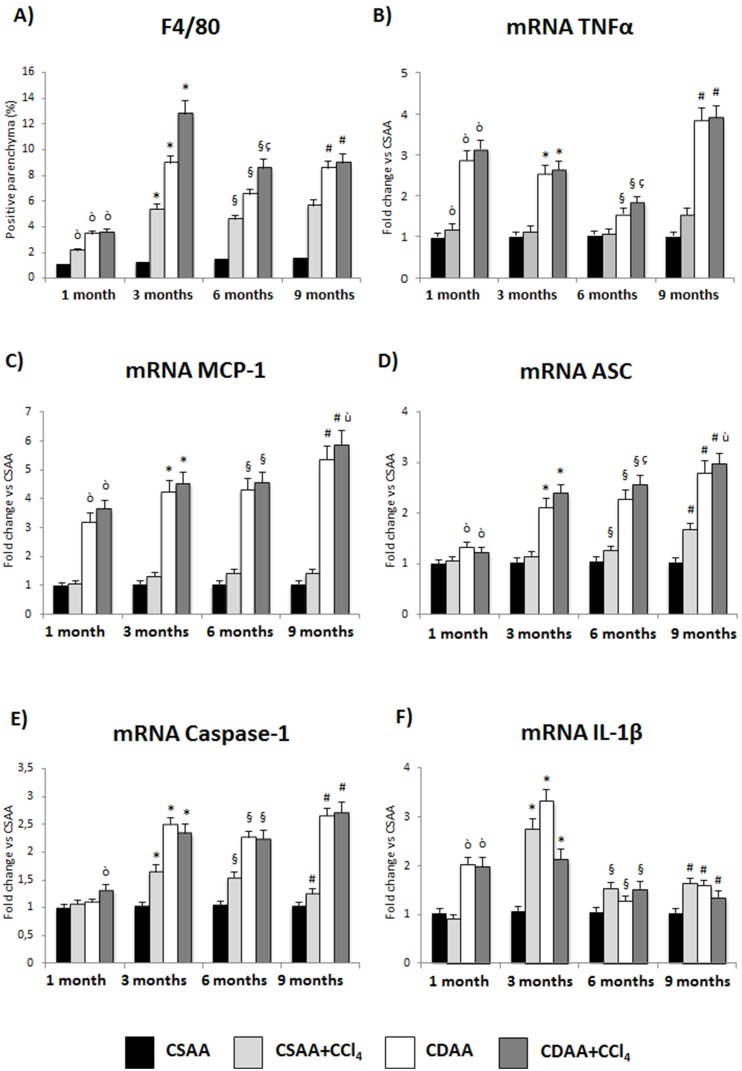
Treatment with CDAA or CDAA+CCl_4_ induces hepatic inflammation in mice. A) Immunohistochemistry for pan-macrophage marker F4/80. B) TNFα and (C) MCP-1 mRNA levels are increased in CDAA or CDAA+ CCl_4_ already after 1 month of treament. D–F) Markers of Inflammasome activation ASC, Caspase-1 and IL-1β show increased expression in CDAA or CDAA+ CCl_4_ groups. Data represent mean ± SD; ò p<0.05 vs CSAA 1 month; *p<0.05 vs CSAA 3 months; §p<0.05 vs CSAA 6 months; #p<0.05 vs CSAA 9 months; ç p<0.05 vs CDAA 6 months; ù p<0.05 vs CDAA 9 months.

**Figure 5:**
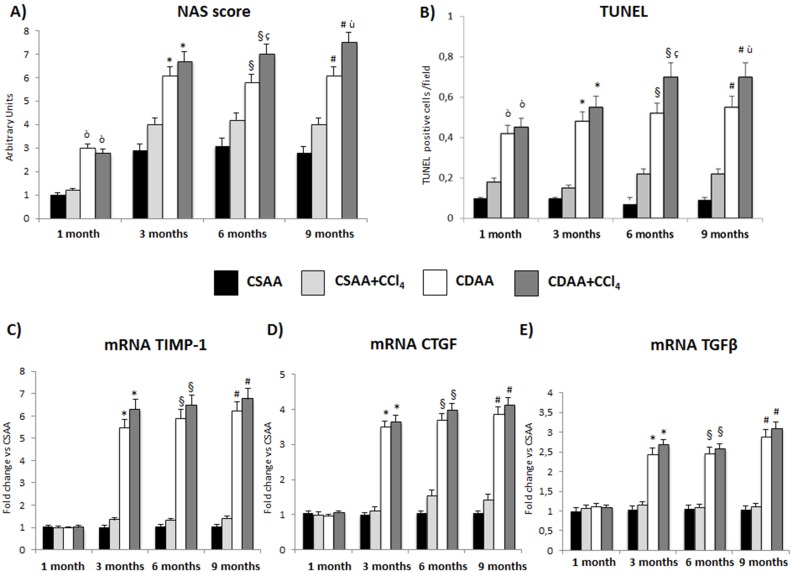
Treatment with CDAA or CDAA+CCl_4_ induces liver injury and expression of markers of hepatic fibrogenesis in mice. A) The total degree of liver injury (hepatic steatosis and necroinflammation) was determined according to the NAS score. B) The number of apoptotic cells was measured with TUNEL and expressed as apoptotic cell/field. C–E) qRT-PCR shows a progressive increase, in a time dependent manner, of the amount of TIMP-1 (C), CTGF (D) and TGFβ (E) mRNA expression in the group of CDAA, with the evidence of a promoting effect exerted by CCl_4_ starting from month 3. Data represent mean ± SD; ò p<0.05 vs CSAA 1 month; *p<0.05 vs CSAA 3 months; §p<0.05 vs CSAA 6 months; #p<0.05 vs CSAA 9 months; ç p<0.05 vs CDAA 6 months; ù p<0.05 vs CDAA 9 months.

**Figure 6:**
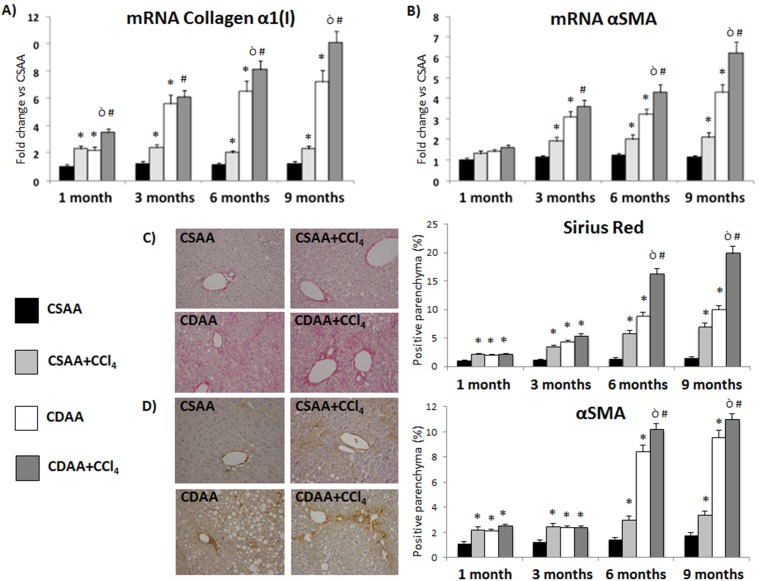
Treatment with CDAA or CDAA+CCl_4_ induces hepatic fibrosis in mice. A–B) qRT-PCR expression shows a progressive increase, in a time dependent manner, of the amount of Collagen α1(I) (A) and αSMA (B) mRNA in the group of CDAA, with the evidence of a promoting effect exerted by CCl_4_ starting from month 6. Conversely, mice treated with control diet (CSAA) do not show any time-dependent increase neither in the level of Collagen α1(I) nor of αSMA, not even after chronic CCl_4_ injection. C–D) Morphometric analysis shows increased collagen deposition by Sirius Red staining (C) and increased HSC activation by αSMA Immunohistochemistry (D) in the liver of CDAA treated mice in comparison to CSAA treated mice. This condition is even enhanced in the liver of mice treated with CDAA+CCl_4_. Data represent mean ± SD; *p<0.05 vs CSAA; #p<0.05 vs CSAA+ CCl_4_; ò p<0.05 vs CDAA.

The effect of CDAA diet in terms of liver injury and fibrosis was analyzed at the level of both gene expression and histological analysis for different aspects of liver injury, inflammation, and fibrosis (Fig. 4–6).

Concerning inflammation, at the histological level, by Immunohistochemistry, the pan-macrophage marker F4/80 increased at all time points tested, reaching the highest level in CDAA+CCl_4_-treated animals at 3 months (Fig. 4A). In addition, CDAA feeding increases at early stage the expression of genes related to liver injury and inflammation, such as the Tumor Necrosis Factor Alpha (TNFα) (Fig. 4B), the Monocyte Chemotactic Protein-1 (MCP-1) (Fig. 4C) and the Inflammasome components as the Apoptosis-associated Speck-like protein containing a CARD (ASC), Caspase-1 and Interleukin-1 beta (IL-1β) (Fig. 4D–F).

After blind evaluation by an expert liver pathologist (M.G.), treatment with CSAA+CCl_4_ induces different degrees of hepatocyte necrosis and lobular inflammation. A more severe degree of steatosis and necrosis is observed at histology in mice treated with CDAA+CCl_4_. The CDAA+CCl_4_ treatment, already at 3 months, is able to create a condition that resembles the human NASH with a NAS score >5 (Fig. 5A). This was associated to a light but significant increase in the number of apoptotic cells as measured by TUNEL staining (Fig. 5B). We further investigated the expression of fibrogenic markers at each time point. CDAA+CCl_4_ treatment induced a stable and significant increase of the Metallopeptidase Inhibitor-1 (TIMP-1) (Fig. 5C) and cytokines involved in collagen synthesis stimulation such as the Connective Tissue Growth Factor (CTGF) and the Transforming Growth Factor β (TGFβ) starting at 3 months (Fig. 5D–E). Notably, CDAA+CCl_4_ treatment shows the higher increase in mRNA expression of Collagen α1(I) in a time-dependent manner (Fig. 6A). Additionally, qRT-PCR analysis shows in the CDAA+CCl_4_ group a time-dependent increase of mRNA levels of αSMA, a marker of Hepatic Stellate Cell (HSC) activation and fibrogenesis, reaching the highest levels at 9 months of treatment (Fig. 6B). The CDAA diet alone is also able to induce fibrogenesis, but the increase is significantly lower than in the CDAA+CCl_4_ group. Conversely, the CSAA diet determined a modest increase in fibrogenesis only in the presence of CCl_4_ (Fig. 6A–B).

Similar results were obtained from the morphometric analysis of the Sirius Red-positive parenchyma that reveals at 6 months the higher collagen deposition in the liver of mice treated with CDAA+CCl_4_ (Fig. 6C). The CDAA diet alone is able to induce collagen deposition, even if at lower levels compared to the CDAA+CCl_4_ group. More importantly, only low collagen deposition is observed in mice subjected to CCl_4_ and treated with CSAA diet. Similar data were obtained by αSMA Immunohistochemistry, which shows the highest level of myofibroblast activation in the group of mice treated with CDAA+CCl_4_ at 6 and 9 months (Fig. 6D).

### Treatment with CDAA+CCL_4_ Induces HCC Development after 9 Months

No tumor development was observed in mice treated with CSAA diet up to 9 months, not even when associated to CCl_4_ injection.

**Figure 7:**
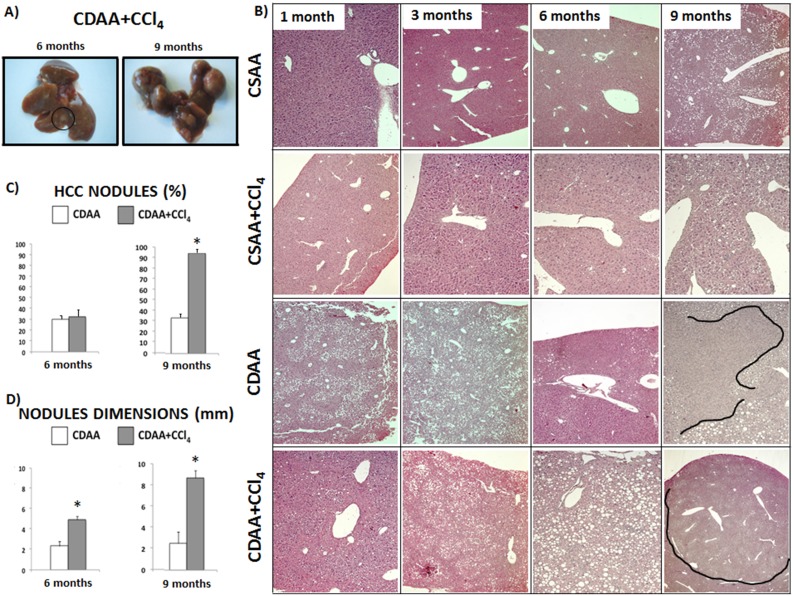
Treatment with CDAA+CCl_4_ induces development of HCC after 9 months. **A**) Mice sacrificed after 1 and 3 months from the beginning of the treatment do not show any nodular lesions in the parenchyma. Conversely, mice sacrificed after 6 months of treatment show few nodular lesions compatible with HCC. After 9 months of treatment mice show a liver parenchyma completely damaged. B) Histochemical staining for H&E and Sirius Red show respectively no fat deposition and absence of collagen content into the nodules. C) After 6 months of treatment, about 30% of mice show HCC development both with CDAA and CDAA+CCl_4_. After 9 months of treatment, 100% of mice from the CDAA+CCl_4_ group show at least one nodule into the liver parenchyma. D) After 6 months, the average of nodules dimension in the group of CDAA mice is significantly lower in comparison to that of the group of CDAA+CCl_4_ mice. After 9 months, the average of nodules dimension in the group of CDAA+CCl_4_ mice results of 9 mm, in comparison to 3 mm of the group of mice treated with CDAA alone. Data represent mean ± SD; *p<0.05 vs CDAA.

At 6 months of treatment, almost 30% of mice treated with CDAA and CDAA+CCl_4_ diets develop at least one nodule of tumor in the liver, with no statistically significant differences between the two groups (Fig. 7A–C). Although there are no differences at 6 months of treatment in the percentage of mice developing tumors, significant differences are observed in the size of the tumors (Fig. 7D).

Notably, after 9 months of treatment, all mice from CDAA+CCl_4_ group show the presence of at least one tumor nodule, with a median dimension of 9 mm. On the other hand, CDAA-fed mice with no CCl_4_ support show HCC in only 35% of cases with a significantly lower average diameter (Fig. 7C–D).

**Figure 8:**
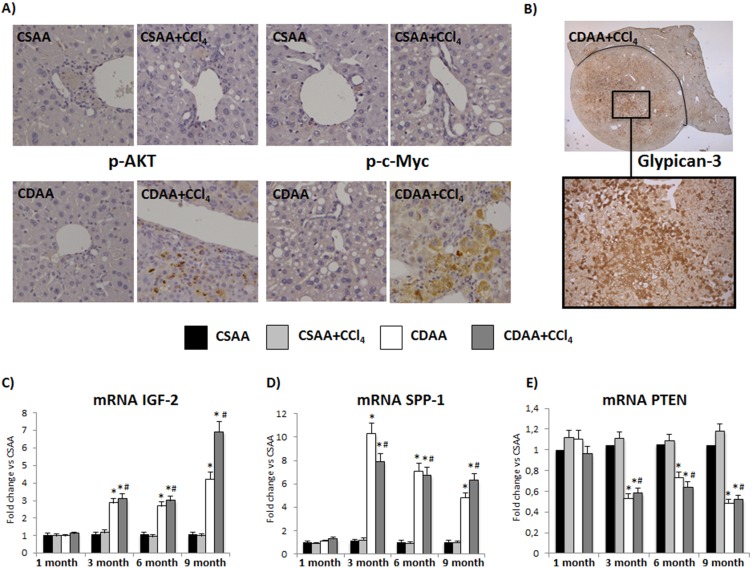
The expression of pro-carcinogenic genes is modified already after 3 months of treatment. A) Immunohistochemistry for p-AKT and p-c-Myc shows positive staining in the liver parenchyma of CDAA+CCl_4_ mice, in correspondence of HCC foci (20X magnification) B) Glypican-3 Immunohistochemistry shows positive staining in the tumor area (5X and 20X magnification). C) Mice treated with CDAA or CDAA+CCl_4_ show, starting at 3 months, increased levels of mRNA for IGF-2 and (D) SPP-1 and (E) decreased mRNA levels of PTEN. Data represent mean ± SD; *p<0.05 vs CSAA; #p<0.05 vs CSAA+CCl_4_.

Microscopically, cancer areas appear completely free from collagen deposition (Fig. 7B). Furthermore, Immunohistochemical evaluation of p-AKT, p-c-Myc and Glypican-3, that are associated to diagnosis and classification of human HCC, reveals a positive staining also in our mouse model [17]–[19]. Specifically, after 9 months of treatment a positive staining for p-AKT and p-c-Myc in the areas of liver parenchyma that were recognized as HCC, was evident in the CDAA+CCl_4_ group, differently from all the other groups (Fig. 8A). Glypican-3 Immunohistochemistry reveals positive staining in the tumor nodules, similarly to what happens in humans, and in the zone 3 of the parenchyma of damaged liver in the CDAA+CCl_4_ group [20] (Fig. 8B). In addition to these data, we did not observe any nuclear staining for β-Catenin (data not shown).

### Changes in Pro-carcinogenic Gene Expression Anticipate Tumor Formation

In CDAA and CDAA+CCl_4_ groups we observed a significant increase in the mRNA expression starting at 3 months of the pro-carcinogenic genes such as the Insulin-like Growth Factor-2 (IGF-2) and Osteopontin (SPP-1), respectively involved in angiogenesis and migration/metastasis (Fig. 8C–D). In the same groups, the expression of the tumor suppressor gene Phosphatase and Tensin Homolog (PTEN) is significantly reduced again starting at 3 months (Fig. 8E). The expression of pro-carcinogenic genes was associated to a progressive increase in hepatocyte proliferation in CDAA+CCl_4_ group as measured by Ki67 labeling that significantly increased at 6 months and reached the highest levels at 9 months (3.8±0.5 labeled parenchymal cell/field vs 2.2±0.5 in CDAA-treated animals, p<0.05 and data not shown).

## Discussion

In this study, we have provided evidences that CDAA diet induces peripheral insulin resistance already in the first month after treatment, and this was associated to the pathological spectrum of NAFLD, including NASH and HCC. Peripheral insulin resistance is a primary feature of NAFLD/NASH, and is probably one of the main co-factors involved in the worsening of the disease [8], [21]. Thus the novelty of our results is the demonstration that the CDAA+CCl_4_ model determines peripheral insulin resistance, NAFLD and its progression to HCC. Using this novel experimental approach we observed: a) development of peripheral insulin resistance already after 1 month; b) entire spectrum of lesions ranging from simple steatosis to NASH and HCC; c) development of HCC after 9 months of treatment in all mice; d) association of HCC development to increased extracellular matrix deposition; e) significant modification of oncogenic genes expression already after 3 months of treatment. Thus, this experimental model is able to guarantee in 9 months the development of HCC in almost 100% of animals and to early resemble the main features of the progression from NAFLD to NASH and HCC.

In the majority of human cases, HCC arises in patients with advanced chronic liver injury and/or cirrhosis [1]. NAFLD, which is present in up to 90% of all obese persons and in up to 70% of persons with type 2 diabetes, is a recognized risk factor for HCC, that may develop in NASH in the absence of cirrhosis [1]. However, the study of the molecular mechanisms linking steatosis development to chronic liver injury and HCC is hampered by the lack of adequate experimental models that often do not resemble the human situation [22], either are not associated to a significant development of chronic liver injury or lead to a cachectic phenotype that does not allow a long period of observation, as needed for carcinogenesis. In the CDAA model, mice develop steatosis in the absence of a high fat diet, mice continue to eat, do not reduce the appetite and the amount of calories introduced and weight changes are similar to control diet. Furthermore, in comparison to other existing rodent models, CDAA is able to drive the progression of steatosis (NAFLD) toward a condition of inflammation and fibrosis (NASH).

We have investigated the potential mechanisms that could explain the hepatic steatosis. First of all we demonstrated that CDAA-treated mice were more insulin resistant (in the periphery) already at one month as compared to the control CSAA diet-treated mice. Data in the literature show controversial results concerning the potential condition of insulin resistance in the course of CDAA treatment in rodents [23], [24], mainly based on methods that provide only a partial and indirect measurement of insulin resistance, and related to the fasting glycemic and insulinemic state [24], [25]. Here insulin resistance was measured by the euglycemic-hyperinsulinemic clamp, which represents the gold standard for the evaluation of insulin sensitivity [26], and the results were confirmed by finding increased fasting insulin concentrations (Figure 1). This condition was even enhanced by the addition of CCl_4_ to the diet. The mechanisms by which CDAA diet induces insulin resistance are unknown but could be related to the gut microbiota metabolism of choline as shown in humans [27]. Metabolomics data have indicated that reduced concentrations of lysophosphocholine, in particular reduced lyso-PC C18∶2, and lyso-PC C16∶0, are associated with peripheral insulin resistance [28] and hepatic steatosis [29], [30] [31]. Moreover, we found that CDAA diet increases Inflammasome components in the liver, which supports our hypothesis of a possible link between gut microbiota modifications, insulin resistance and progression of liver injury [32], [33]. The discovery that CDAA diet induces peripheral insulin resistance is important for the translation of this animal model to the human studies. To our knowledge, this is the first experimental model where a clear link between peripheral insulin resistance, NASH development and HCC formation has been established. In human patients with NAFLD/NASH, peripheral insulin resistance is a primary feature of the disease, even in lean subjects that do not present the characteristics of metabolic syndrome [34]. Moreover, a worse peripheral insulin resistance state has been associated with the presence of fibrosis [8]. To investigate if CDAA was worsening also hepatic insulin resistance, we have measured the expression of liver enzymes associated with glucose production (G6Pase), gluconeogenesis (PEPCK) and de-novo lipogenesis as SREBP-1c and ChREBP [16], [35]. The results show that G6Pase and PEPCK mRNA expression was decreased, indicating a parallel decrease in glucose production. This is in agreement with the observed reduction in FPG and possibly due to the increase in fasting plasma insulin that is known to suppress both glucose production and gluconeogenesis [36]. ChREBP and SREBP-1c expressions were also reduced indicating that de-novo lipogenesis was not directly implicated in the development of liver steatosis. On the other hand, the reduction in ACOX-1 and CPT1A indicates that CDAA diet reduced hepatic fatty acid oxidation and this could be one of the mechanisms for hepatic triglyceride deposition and for the higher lipid deposition in CDAA+CCl_4_ treated animals, presumably mediated by TGFβ signaling in hepatocytes [12] [37]. Thus, we can conclude that CDAA induces steatosis mainly by reducing FFA oxidation, while neither de novo lipogenesis nor glucose production seem to play an important role.

Despite the intrinsic differences among etiological factors for HCC, a common denominator at the origin of this neoplasia is the perpetuation of a wound-healing response triggered by parenchymal cell death, the ensuing inflammatory reaction and the concomitant fibrosis progression [38]. Indeed, HCC almost always develops on a background of chronic liver injury including chronic hepatitis and cirrhosis, conditions referred as preneoplastic stages [39]. Accumulating evidences indicate that the inflammatory reaction characteristic of chronic liver injury actively participates in the development of hepatic fibrosis, as well as in the activation of the potent regenerative response of liver parenchyma, ultimately leading to HCC development [40], [41]. For instance, the production of cytokines such as TNFα and IL-6 is essential to trigger hepatocyte proliferation, liver regeneration and animal survival after partial hepatectomy [41]. On this regard, in our model we observed an higher number of infiltrating macrophages (significantly increased in CDAA+CCl_4_-treated mice compared to all other groups) and this was accompanied by a complete repertoire of the inflammatory response such as apoptotic cells, TNFα, MCP-1 and components of the Inflammasome pathway (ASC, Caspase-1, IL-1β) gene expression. Inflammasomes are intracellular multiprotein complexes expressed in both parenchymal and non-parenchymal cells of the liver that in response to cellular danger signals activate Caspase-1 and release the pro-inflammatory cytokine IL-1β, and their role in NASH development is controversial [42]. In a majority of Inflammasomes, activation of Caspase-1 requires interaction with the adaptor protein ASC and this leads to increased IL-1β synthesis and secretion, that finally synergizes with TNFα to induce cytotoxicity, HSC activation and maintenance of macrophages in inflammatory state [43]. Thus, it is possible that the inflammatory milieu occurring in the damaged liver in our model favors survival of preneoplastic hepatocytes, promotes the generation of a fibrotic substrate and eventually contributes to carcinogenesis [44].

As known from the literature, both hepatic steatosis and fibrosis represent independent risk factors for HCC development [45], [46]. In our hands, HCC development was associated to steatosis and fibrosis in 35% of mice in the CDAA alone-treated group. Recent studies used CDAA diet in mice: Denda et al. reported a 100% incidence in preneoplastic foci after 65 weeks of treatment, with no further information on the presence of lesions at earlier time points [47]. In the study from Kodama et al. mice fed CDAA diet up to 20 weeks showed steatosis and fibrosis, with no mention on the development of carcinogetic foci [23]. In our study the addition of a low-chronic dose of CCl_4_ to the CDAA administration promoted HCC development in 100% of mice after 9 months of treatment. This was associated to increased extracellular matrix deposition. Thus, CCl_4_ should be considered as a promoting factor in CDAA diet-induced liver damage. It could be hypothesized that, even at low doses, CCl_4_ synergizes with hepatic steatosis in the development of liver injury and HCC, as shown by increased fibrosis deposition at 6 months and increased nodules formation at 9 months in CDAA+CCl_4_-treated mice. On this regard, in our model we observed higher macrophages infiltration at 3 months in CDAA+CCl_4_-treated animals, followed by increased liver injury (as determined by the NAS score), markers of fibrogenesis (type I collagen gene expression and HSC activation) and extracellular matrix deposition starting from 6 months in CDAA+CCl_4_-treated mice.

Thus our model shows all the main features of the typical condition of HCC development, including the early modification in the expression of pro-carcinogenetic genes, increased hepatocyte proliferation and the expression of markers in the tumor parenchyma, similarly to the human condition. Specifically, both in the CDAA and CDAA+CCl_4_ groups increased mRNA levels of IGF-2 and SPP-1 genes, respectively involved in angiogenesis and migration/metastasis [48], and decreased mRNA levels of the oncosoppressor gene PTEN were observed at 3 and 9 months [49], [50], additionally confirming the neoplastic potential of our model even at early stages. In addition to the current data, several similarities with the human pattern of HCC have been shown in our model, by the positive staining for p-AKT, p-c-Myc and Glypican-3 distribution observed in the tumor parenchyma of mice treated for 9 months with CDAA and CCl_4_. Furthermore, the absence of any nuclear stain for β-Catenin, which is usually observed in poorly differentiated HCC is consistent with the histopathological diagnosis of well differentiated HCC in our mouse model.

In conclusion, our study provides a reproducible model that recapitulate the pathological spectrum of human NALFD evolving towards HCC, highlighting the potential role of peripheral vs hepatic insulin resistance and may therefore represent an ideal tool to investigate the interaction mechanisms and new potential genes hypothetically involved in HCC development.
